# Prognostic Characteristics of MACC1 Expression in Epithelial Ovarian Cancer

**DOI:** 10.1155/2018/9207153

**Published:** 2018-11-01

**Authors:** Hoiseon Jeong, Jiyoon Jung, Hwa Eun Oh, Jung-Woo Choi, Eung Seok Lee, Young-Sik Kim, Ju-Han Lee

**Affiliations:** Department of Pathology, Korea University Ansan Hospital, Ansan, Republic of Korea

## Abstract

Recent studies have shown that overexpression of metastasis-associated in colon cancer 1 (MACC1) is significantly associated with adverse prognoses of patients with different kinds of cancer. However, the exact survival effect of MACC1 on epithelial ovarian cancer (EOC) patients has not yet been established. Thus, the objective of this study was to explore the prognostic role of MACC1 mRNA in EOC by using Kaplan-Meier (KM) plotter and ONCOMINE database. Our results indicated that MACC1 mRNA high expression was significantly associated with unfavorable overall survival (hazard ratio (HR) = 1.51 (95% confidence interval (CI): 1.21 – 1.88),* P* = 0.00025) and progression-free survival (HR = 1.53 (95% CI: 1.24 – 1.89),* P* = 5.8e-05) in EOC patients. We also found that the expression of MACC1 mRNA in EOC was 2.5 times higher than that in normal surface ovarian epithelium, which was statistically significant (*P* = 2.86e-7). Our results suggest that MACC1 expression might be a biomarker for poor prognosis in individual EOC patients.

## 1. Introduction

It has been estimated that there will be about 22,240 new ovarian cancer patients diagnosed and approximately 14,070 ovarian cancer deaths in the US in 2018 [1]. Among gynecological cancers, epithelial ovarian cancer (EOC) causes the majority of cancer-related deaths, despite recent development of treatment. There is about an 80% of tumor recurrence in stage III or IV cases of EOC [2]. Therefore, it is critical to identify prognostic biomarkers that can predict the survival prognosis of individual EOC patients.

Metastasis-associated in colon cancer 1 (MACC1) gene, a novel regulator of tumor growth and metastasis, has recently been identified in colon cancer [3]. MACC1 controls hepatocyte growth factor (HGF)/Met signaling pathway and enhances migration, invasion, and metastasis of cancer cells [3, 4]. Some studies have suggested that MACC1 overexpression is significantly associated with unfavorable clinical outcomes in various cancers [5–9]. However, the precise survival effect of MACC1 on EOC patients remains unclear.

Kaplan-Meier (KM) plotter (www.kmplot.com) can assess the genetic effect on survival. Gene expression data and overall and progression-free survival information can be downloaded from “Gene Expression Omnibus (GEO),” “The Cancer Genome Atlas (CGA),” and “The European Genome-phenome Archive” [10]. Especially, Gene Expression Omnibus (GEO) is a public repository for genomic data supported by the National Center for Biotechnology Information (NCBI) that, currently, contains nearly two million samples [11]. The KM plotter database includes gene expression and survival information of 1,816 EOC patients. ONCOMINE (www.oncomine.org) is a cancer microarray data-mining platform for differential gene expression between cancer and normal tissue [12]. The objective of this study was to determine the prognostic role of MACC1 expression in EOC patients by using KM plotter and ONCOMINE database.

## 2. Materials and Methods

### 2.1. Kaplan-Meier (KM) Plotter

The prognostic value of MACC1 mRNA transcription level was measured using the KM plotter, an online open database consisting of gene expression profiles and survival information for ovarian cancer patients. Using this database, MACC1 mRNA transcription level was only measured by HG-U133 Plus 2.0 platform. Recently, HG-U133 Plus 2.0 platform is the most popular and high accurate method among microarray platforms. It contains 54,220 probes [11].

Nine GEO datasets out of 15 KM plotter datasets were used to determine the association between MACC1 mRNA expression level and survival outcomes as follows: Gene Expression Omnibus Series (GSE) 9891 (n = 285) [13], GSE 26193 (n = 107) [14], GSE 63885 (n = 101) [15], GSE 30161 (n = 58) [16], GSE 18520 (n = 63) [17], GSE 27651 (n = 49) [18], GSE 19829 (n = 28) [19], GSE 65986 (n = 55) [20], and GSE 51373 (n = 28) [21]. A total of 774 patients were used in the present analysis.

These patients were divided into two groups based on the expression of MACC1. Patients with higher MACC1 expression than the median separated were pooled into the group with high expression, while those with MACC1 expression lower than the median separated were pooled into the group with low expression. Other statistical outcomes, including hazard ratio (HR), 95% confidence intervals (CI), and log rank P calculated from the database, were also included in the figures and tables of this manuscript. Values of* P* < 0.05 were used to indicate statistically significant difference.

### 2.2. ONCOMINE Data-Mining Analysis

ONCOMINE, an online web-based cancer database for RNA and DNA sequences, was used to facilitate data mining of transcriptional expression of MACC1 in ovarian cancer. Data of Lu et al. [22] were used. Transcriptional expression of MACC1 in cancer samples was compared with that in normal surface ovarian epithelium samples using Student's* t*-test. Statistically significant values and fold change were demarcated as *P* < 0.05 and 2, respectively.

## 3. Results

The prognostic value of MACC1 in the database was determined by using KM plotter. Affymetrix ID was valid 1566766_s_at (MACC1). Among 774 patients, overall survival (OS) and progression free survival (PFS) were available for 655 and 614 patients, respectively.

**Figure 1:**
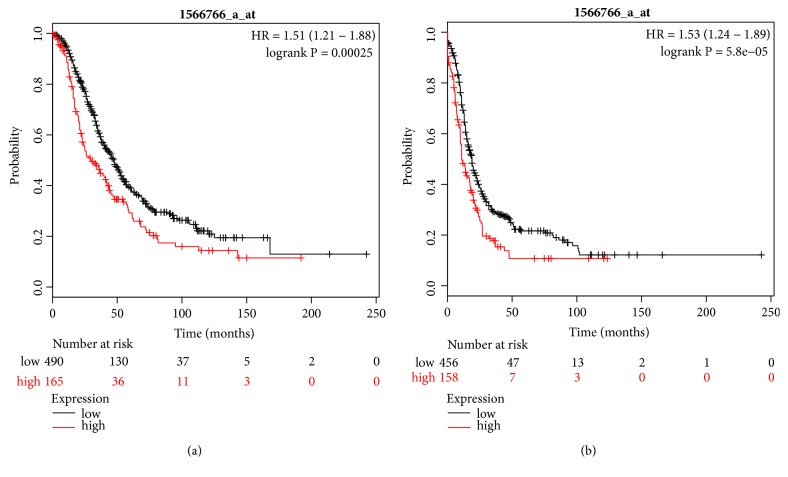
Prognostic value of MACC1 mRNA expression in the database. Affymetrix ID is valid 1566766_s_at (MACC1). (a) Overall survival curves are plotted for all epithelial ovarian cancer patients (n = 655). (b) Progression-free survival curves are plotted for all epithelial ovarian cancer patients (n = 614).

KM plot curves showed that high expression of MACC1 mRNA was significantly correlated with poor OS in 655 patients with EOC (HR = 1.51 (95% CI: 1.21 – 1.88),* P* = 0.00025) (Figure 1(a)). Of these 655 OS available EOC patients, 553 and 570 cases were able to analyse histologic type [serous carcinoma (n = 523) and endometrioid carcinoma (n = 30)] and clinical stages [stage I or II (n = 83) and III or IV (n = 487)], respectively. MACC1 mRNA high expression was significantly correlated with poor OS in 523 cases with serous carcinoma (HR = 1.48 (95% CI: 1.16 – 1.89),* P* = 0.0014) and in 30 patients with endometrioid carcinoma (HR = 7.31 (95% CI: 0.76 – 70.29),* P* = 0.043). In addition, high expression of MACC1 mRNA was significantly associated with poor OS in 487 patients with stage III or IV EOC (HR = 1.56 (95% CI: 1.22 – 1.99),* P* = 0.00029) (Table 1). However, high expression of MACC1 mRNA was not significantly associated with OS in 83 patients with stage I or II EOC (Table 1).

KM plot curves showed that high expression of MACC1 mRNA was significantly correlated with unfavorable PFS in 614 EOC patients (HR = 1.53 (95% CI: 1.24 – 1.89),* P* = 5.8e-05) (Figure 1(b)). Of these 614 PFS available EOC patients, 527 and 609 patients were able to be analysed for histologic type [serous carcinoma (n = 483) and endometrioid carcinoma (n = 44)] and clinical stages [stage I or II (n = 115) and III or IV (n = 494)], respectively. MACC1 mRNA high expression was significantly related to poor PFS in 483 serous carcinomas (HR = 1.45 (95% CI: 1.16 – 1.82), *P* = 0.0013) and in 494 patients with stage III or IV (HR = 1.50 (95% CI: 1.21 – 1.86),* P* = 0.00024) (Table 2). However, the high expression of MACC1 mRNA was not statistically significant in 44 patients with endometrioid cancer and in 115 patients with stage I or II ovarian cancer patients (Table 2).

**Figure 2:**
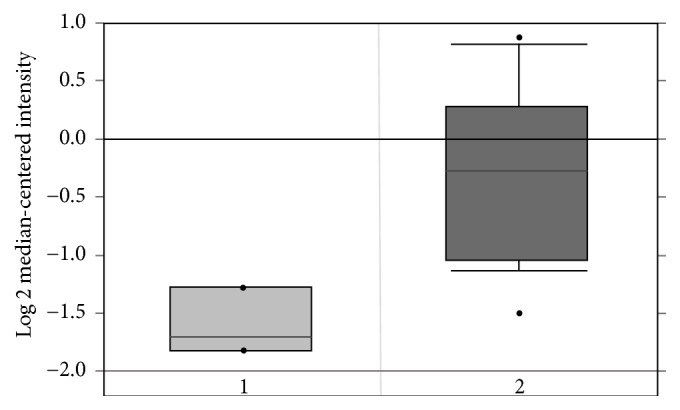
Transcriptional level of MACC1 mRNA in normal ovarian tissue (1) and epithelial ovarian cancer (2). The fold change is 2.543 (p = 2.86e-7).

Using the ONCOMINE database, we measured MACC1 mRNA expression between EOC and normal ovarian tissue. Twenty serous ovarian carcinoma samples and five normal surface ovarian epithelial tissues were used. The average fold change was 2.543, which was statistically significant (*P* = 2.86e-7) (Figure 2).

## 4. Discussion

Results of this study revealed that high expression of MACC1 mRNA was significantly associated with unfavorable OS and PFS of patients with EOC. In addition, MACC1 mRNA levels in EOC were significantly higher than those in normal surface ovarian epithelium.

Most EOC patients present with advanced stage disease (stage III or IV) due to the lack of early symptoms [2]. The five-year OS rate for this group has remained to be lower than 30%, despite aggressive treatment, including debulking surgery and combination chemotherapy [2]. No certified prognostic biomarkers have been established for individual EOC patient.

MACC1 was newly identified as an oncogene that controls hepatocyte growth factor/Met pathway and promotes cancer cell migration and invasion in both cell cultures and xenograft models [3, 4]. Many studies have demonstrated that MACC1 overexpression is correlated with worse clinical outcomes in cancer patients [5–9]. A recent meta-analysis has shown that high expression of MACC1 is significantly associated with poor OS of patients with multiple solid tumors (HR = 2.11 (95% CI: 1.59 – 2.80),* P* < 0.001) [23]. However, there has been a lack of studies on MACC1 expression in EOC. A few studies have used immunohistochemical staining method to investigate the expression of MACC1 protein in EOC patients and clinicopathologic parameters. Li et al. [24] have reported that positive expression of MACC1 protein of EOC is significantly associated with lymph node metastasis and advanced clinical stages. Yu et al. [25] have shown that positive expression of MACC1 protein is significantly related to lymph node metastasis, implantation, advanced clinical stage, and unfavorable survival outcomes in EOC patients. However, the immunohistochemical staining method may vary depending on the kind of antibody used, tissue fixation time, and data interpretation.

Up to the moment, no study has reported the survival effect of MACC1 mRNA level in EOC patients. Our study showed an approximate 50% decrease in OS or PFS rate in the presence of high MACC1 mRNA level in EOC patients. The value, especially, of MACC1 as a biomarker is more evident in patients with advanced stage EOC than that in those with early stages of EOC. Thus, its clinical utility will be greater for those with advanced stages of EOC. In this study, high expression of MACC1 mRNA in endometrioid cancer patients was significantly associated with unfavorable OS, although PFS did not show statistically significant results. Recently, the interest in carcinogenesis of endometrioid or clear cell carcinoma associated with endometriosis has been increasing [26]. However, this study was limited in the number of endometrioid cancer cases to produce definite conclusions. Further study is needed to determine the possible role of MACC1 depending on histologic type of EOC.

Difficulty in obtaining a normal surface ovarian epithelium was a problem in EOC research. This study compared EOC samples with just pooled scrapings of normal ovarian surface epithelium rather than whole ovarian tissue [22]. This study found that the expression of MACC1 mRNA in EOC was 2.5 times higher than that in normal surface ovarian epithelium. Unfortunately, the number of patients used for this ONCOMINE analyses was too small. Although this study cannot yield a definitive conclusion, MACC1 may have a potential for early detection of EOC.

Recent investigation has indicated that targeting MACC1 may have a therapeutic impact on EOC. Sheng et al. [27] and Zhang et al. [28] have reported that MACC1 specific small interfering RNA and MACC1 specific small hairpin RNA can decrease Met protein expression in EOC cell line, respectively.

## 5. Conclusion

Our results demonstrated distinct prognostic roles of MACC1 mRNA expression in EOC patients and the difference in expression between cancer and normal tissue. Results of this study suggest that MACC1 might be a biomarker for poor prognosis in EOC patients. It may be a potential drug target.

## Figures and Tables

**Table 1:** Correlation of MACC1 mRNA expression between overall survival and clinicopathologic parameters of epithelial ovarian cancer patients.

	Cases	HR	95% CI	p value
Overall	655	1.51	1.21 – 1.88	0.00025
Histologic type*∗*				
Serous	523	1.48	1.16 – 1.89	0.0014
Endometrioid	30	7.31	0.76 – 70.29	0.043
Clinical stages*∗*				
I, II	83	0.53	0.19 – 1.45	0.210
III, IV	487	1.56	1.22 – 1.99	0.00029

*∗*; 553 and 570 out of 655 ovarian cancer cases could be analysed for histologic type and clinical stages, respectively.

**Table 2:** Correlation of MACC1 mRNA expression between progression-free survival and clinicopathologic parameters of epithelial ovarian cancer patients.

	Cases	HR	95% CI	p value
Overall	614	1.53	1.24 – 1.89	5.8e-5
Histologic type*∗*				
Serous	483	1.45	1.16 – 1.82	0.0013
endometrioid	44	0.56	0.19 – 1.61	0.270
Clinical stages*∗*				
I, II	115	0.53	0.26 – 1.09	0.081
III, IV	494	1.50	1.21 – 1.86	0.00024

*∗*; 527 and 609 out of 614 ovarian cancer cases could be analysed for histologic type and clinical stages, respectively.

## Data Availability

The data used to support the findings of this study are available from the corresponding author upon request.
